# Racial and Gender Discrimination Predict Mental Health Outcomes among Healthcare Workers Beyond Pandemic-Related Stressors: Findings from a Cross-Sectional Survey

**DOI:** 10.3390/ijerph18179235

**Published:** 2021-09-01

**Authors:** Rachel Hennein, Jessica Bonumwezi, Max Jordan Nguemeni Tiako, Petty Tineo, Sarah R. Lowe

**Affiliations:** 1 Yale School of Medicine, Yale University, New Haven, CT 06511, USA; 2 Department of Epidemiology of Microbial Diseases, Yale School of Public Health, Yale University, New Haven, CT 06511, USA; 3 Department of Psychology, Montclair State University, Montclair, NJ 07043, USA; bonumwezij1@mail.montclair.edu (J.B.); tineop1@mail.montclair.edu (P.T.); 4 Department of Internal Medicine, Brigham and Women’s Hospital, Boston, MA 02115, USA; max.tiako@yale.edu; 5 Department of Social and Behavioral Sciences, Yale School of Public Health, Yale University, New Haven, CT 06511, USA; sarah.lowe@yale.edu

**Keywords:** discrimination, race and ethnicity, healthcare workers, mental health

## Abstract

Racial and gender discrimination are risk factors for adverse mental health outcomes in the general population; however, the effects of discrimination on the mental health of healthcare workers needs to be further explored, especially in relation to competing stressors. Thus, we administered a survey to healthcare workers to investigate the associations between perceived racial and gender discrimination and symptoms of depression, anxiety, posttraumatic stress, and burnout during a period of substantial stressors related to the COVID-19 pandemic and a national racial reckoning. We used multivariable linear regression models, which controlled for demographics and pandemic-related stressors. Of the 997 participants (Mean Age = 38.22 years, SD = 11.77), 688 (69.01%) were White, 148 (14.84%) Asian, 86 (8.63%) Black, 73 (7.32%) Latinx, and 21 (2.11%) identified as another race. In multivariable models, racial discrimination predicted symptoms of depression (B = 0.04; SE: 0.02; *p* = .009), anxiety (B = 0.05; SE: 0.02; *p* = .004), and posttraumatic stress (B = 0.01; SE: 0.01; *p* = .006) and gender discrimination predicted posttraumatic stress (B = 0.11; SE: 0.05; *p* = .013) and burnout (B = 0.24; SE: 0.07; *p* = .001). Discrimination had indirect effects on mental health outcomes via inadequate social support. Hospital-wide diversity and inclusion initiatives are warranted to mitigate the adverse mental health effects of discrimination.

## 1. Introduction

The COVID-19 pandemic has significantly impacted the mental health of healthcare workers (HCWs) since the outbreak began in 2019. A meta-analysis of 65 studies assessing mental health outcomes among HCWs during the pandemic found that the prevalence of anxiety was 22.1%, depression was 21.78%, and posttraumatic stress disorder was 21.5% [1]. Studies have identified that certain pandemic-related stressors were associated with increased risk of adverse mental health outcomes among HCWs, including work stressors (e.g., frontline status) and social stressors (e.g., childcare shortages) [2].

In addition to these pandemic-related stressors, racial/ethnic minority and female HCWs face stressors related to racial and gender discrimination [3,4], putting them at increased risk for adverse mental health outcomes. For example, a meta-analysis of 293 studies found that racism was associated with psychological distress in the general population [5]. Although studies have explored experiences of racial discrimination among HCWs, those that link racism to mental health outcomes are sparse [6]. Gender discrimination is also salient within hospitals and is associated with adverse mental health outcomes; a survey study identified that 94% of female physicians experienced gender discrimination, which was associated with increased risk of burnout [7].

Identifying factors that link discrimination with mental health outcomes can shed light on how discrimination impacts psychological distress among HCWs and strategies to mitigate harm [8]. For example, the social support deterioration model proposes that discrimination damages social relationships, thereby leading to social isolation and increasing risk for adverse mental health outcomes [9]. A study of Black college students tested this model and found significant indirect effects of racial discrimination on mental health through social support [10]. However, no studies to our knowledge have attempted to test the social support deterioration model among HCWs.

Thus, we conducted a survey study of HCWs to examine the relationships between perceived racial and gender discrimination and symptoms of psychological distress, including depression, anxiety, posttraumatic stress, and burnout. We tested the hypotheses that racial and gender discrimination predicted mental health outcomes above and beyond other pandemic-related stressors, and that discrimination had indirect effects on mental health outcomes through inadequate social support.

## 2. Materials and Methods

### 2.1. Setting

This cross-sectional survey study sought to identify factors associated with mental health among HCWs during the COVID-19 pandemic in the US. We collected data from 1 December 2020 to 14 January 2021. During this period, the incidence of COVID-19 ranged from 139,152 to 314,093 new cases per day [11]. There were 22,645,757 cumulative cases of COVID-19 and 381,552 cumulative deaths due to COVID-19 in the US on the last day of data collection [12].

### 2.2. Recruitment

We sampled hospitals from states with high rates of COVID-19 transmission using a geographic mapping tool that reported transmission data by state [13]. We used convenience sampling by emailing department chairs and affinity group leaders to invite their teams to participate. We required participants to be at least 18 years of age and work at a clinic/hospital, including, but not limited to, physicians, nurses, health technicians, and non-clinical HCWs. We determined our target sample size using methods similar to another study that assessed HCW mental health during the COVID-19 pandemic [14]. We used the formula n = Z**_a_^2^**P(1−P)/d**^2^**, where n is the minimum sample size, P is the prevalence of disease, and d is the precision limit. We used a significance level of alpha = .05 and thus set Z**_a_** = 1.96. We used the estimated prevalence of depression among HCWs during the COVID-19 pandemic, 50.4%, from a prior study that was published shortly before we began data collection [14]. The estimated acceptable precision limit was 0.05. To ensure adequate power for subgroup analyses, we increased the estimated sample size by 50%, leading to a target sample of 576 completed surveys. The Yale Institutional Review Board approved our study procedures. We employed the American Association of Public Opinion Research reporting guidelines [15].

### 2.3. Data Collection Tool

We used a web-based survey to collect responses anonymously, in order to encourage participants to share their experiences honestly. We collected data on racial and gender discrimination, mental health outcomes, pandemic-related social and work stressors, and demographic characteristics.

**Discrimination**. We employed the 18-item General Ethnic Discrimination Scale to assess experienced racism in the past year (i.e., from January 2020–2021) [16]. This scale evaluates the frequency of various experiences of racial/ethnic discrimination. We included an additional item asking respondents how often they have been discriminated against by patients [6]. Each item was scored using a 6-point scale, from “never” (1) to “almost all the time” (6). All items were summed to create a racial discrimination score, ranging from 19 to 114. Previous studies validated this scale for use among Black, Latinx, Asian and White respondents [16]. In our sample, Cronbach’s alpha for the General Ethnic Discrimination Scale was 0.94, indicating high reliability. We also included a single item asking respondents how often they were treated unfairly based on their gender in the previous year (i.e., from January 2020–2021), which was scored using the same 6-point scale as the racial discrimination measure. This item has been used to assess gender discrimination among both men and women [17].

**Mental health outcomes.** We used the 9-item Patient Health Questionnaire (PHQ-9) to measure depressive symptoms [18], 7-item Generalized Anxiety Disorder scale (GAD-7) to assess anxiety symptoms [19], 4-item Primary Care-PTSD scale (PC-PTSD) to assess PTSD symptoms [20], and 2-item Maslach Burnout Inventory to assess burnout symptoms [21]. These measures have been validated for use among HCWs [14,21,22,23,24]. In our sample, Cronbach’s alpha for the PHQ-9 was 0.88, for the GAD-7 was 0.92, for the PC-PTSD was 0.67, and for the Maslach Burnout Inventory was 0.64.

**Pandemic-related social and work factors.** We assessed social support needs using one item from the National Health and Nutrition Examination Survey, which asks respondents if they need a lot, some, a little, or no additional support [25]. We also included dichotomous items indicating any changes in housing during the pandemic and if any family members or close friends were diagnosed with COVID-19. We assessed childcare needs via a single item to which the respondent could respond that they needed a lot, a little, and no more childcare support. For pandemic-related work stressors, we included a question on frontline status, to which respondents could choose that they directly worked with COVID-19 patients (Direct), worked with COVID-19 patients remotely (Indirect), or did not work with COVID-19 patients (None). We also included items on changes in work roles (Yes/No) and changes in work hours (More, Less, or Same).

**Demographic characteristics.** We collected data based on previous studies that identified certain HCW characteristics to be associated with mental health outcomes, including age, gender, race, ethnicity, marital status, household income, and profession [1,2,26,27]. Given studies that identified HCWs with preexisting mental health diagnoses to be at heightened risk for adverse mental health outcomes during the COVID-19 pandemic [26], we also collected data on pre-pandemic mental health diagnoses.

### 2.4. Data Analysis

First, we computed descriptive statistics for the sample. We conducted a missing data analysis by comparing descriptive statistics of the included respondents with those who dropped out due to missing data using independent-samples t-tests and chi-square analysis. We used one-way analysis of variance (ANOVA) and Bonferroni-corrected post-hoc tests to assess differences in racial discrimination by race and ethnicity and gender discrimination by gender. Four participants who identified as non-binary or transgender were dropped due to insufficient statistical power to assess differences by gender minority status. Next, we used unadjusted and adjusted linear regression models to evaluate the associations between racial and gender discrimination and symptoms of depression, anxiety, posttraumatic stress, and burnout. We included demographic characteristics and pandemic-related social and work stressors in the models to determine if discrimination was associated with mental health outcomes beyond competing stressors. We also tested if the three-way interaction between race (non-White vs. White), gender (female vs. male), and discrimination was associated with mental health outcomes based on previous findings that discrimination impacts people differentially by both race and gender [6,8].

In secondary analyses, we tested the hypothesis that adverse effects of racial and gender discrimination on mental health were indirect through inadequate social support. This hypothesis is based on previous qualitative studies that identified racial and gender discrimination among HCWs to lead to lower levels of perceived social support [28,29,30] and a quantitative study that identified significant indirect effects of discrimination on mental health outcomes through social support [10]. This analysis assumes that discrimination precedes inadequate social support, which is a reasonable assumption given studies that have identified discrimination to occur across the life course, from childhood to adulthood [8]. The analysis also assumes that inadequate social support precedes mental health outcomes, which is reasonable based on numerous studies that identified lack of social support as a risk factor for adverse mental health outcomes in both cross-sectional and longitudinal studies (i.e., social causation) [31]. Thus, we aimed to test the pathway through which racial and gender discrimination increase the risk for inadequate social support (assessed using the social support needs indicator), which then increases risk for adverse mental health outcomes. Analyses were conducted in SPSS 27.0 (IBM Corp., 2020) and the PROCESS macro was used to run the interaction analyses (Model 1) and indirect effects analyses (Model 4) [32]. Indirect effects were considered statistically significant when their 95% confidence interval (95%CI) did not include zero. For all other analyses, we considered *p* < .05 to be statistically significant.

## 3. Results

### 3.1. Study Sample

Table 1 presents descriptive statistics for our sample. Of the 997 HCWs, 688 (69.01%) were White, 148 (14.84%) were Asian, 86 (8.63%) were Black, 73 (7.32%) were Latinx, and 21 (2.11%) identified as another race including American Indian/Alaska Native, Native Hawaiian/Pacific Islander, and unspecified. Most of our sample included female HCWs (*n* = 712, 71.41%) and 258 (25.88%) were parents to at least one child who required childcare. The mean age was 38.22 years (SD = 11.77). There were no significant differences between HCWs who were included in the analytic sample (*n* = 997) and those who were dropped due to missing data (*n =* 56).

The mean scores for racial and gender discrimination stratified by race/ethnicity and gender, respectively, are depicted in Table 1. Using Bonferroni-corrected post-hoc tests, racial discrimination scores were significantly higher among Asian (mean difference [M_diff_] = 6.06; standard error difference [SE_diff_] = 0.66; *p* < .001), Black (M_diff_ = 12.75; SE_diff_ = 0.83; *p* < .001), and Latinx (M_diff_ = 5.99; SE_diff_ = 0.91; *p* < .001) HCWs compared with White HCWs. Black HCWs had significantly heightened discrimination experiences compared with Asian (M_diff_ = 6.70; SE_diff_ = 0.98; *p* < .001) and Latinx HCWs (M_diff_ = 6.76; SE_diff_ = 1.16; *p* < .001). Gender discrimination scores were significantly higher among female, compared with male, HCWs (M_diff_ = 0.69; SE_diff_ = 0.06; *p* < .001).

### 3.2. Predictors of Mental Health Outcomes

Factors predicting mental health outcomes using unadjusted and adjusted models are summarized in Table 2 and Table 3, respectively. We did not identify significant interaction effects of race, gender, and racial discrimination on any mental health outcomes, or race, gender, and gender discrimination on any mental health outcomes (results available upon request).

**Depressive symptoms.** In unadjusted models, perceived racial discrimination (unstandardized beta coefficient [B] = 0.07; *p* < .001) and gender discrimination (B = 0.91; *p* < .001) were associated with depressive symptoms. The fully adjusted model predicted 28.73% of the variance in depressive symptoms. Self-reported racial discrimination, but not gender discrimination, was significantly associated with higher depressive symptoms in the adjusted model (B = 0.04; *p* = .033). Other significant predictors of higher depression included greater social support needs, older age, having a pre-pandemic mental health diagnosis, and working more hours during the pandemic compared with no change in work hours. Black HCWs had significantly less severe depressive symptoms compared with White HCWs after controlling for discrimination and other covariates.

**Anxiety symptoms**. Perceived racial discrimination (B = 0.07; *p* < .001) and gender discrimination (B = 0.90; *p* < .001) predicted anxiety symptoms in unadjusted models. The fully adjusted model explained 31.52% of the variance in anxiety symptoms. Racial discrimination, but not gender discrimination, remained a significant predictor of higher anxiety symptoms in the adjusted model (B = 0.05; *p* = .004). Having greater social support needs, a pre-pandemic mental health diagnosis, a family member diagnosed with COVID-19, and certain professions were predictive of higher anxiety. Black HCWs had significantly lower anxiety symptoms compared with White HCWs.

**Posttraumatic stress symptoms**. In unadjusted models, perceived racial discrimination (B = 0.03; *p* < .001) and gender discrimination (B = 0.31; *p* < .001) were associated with posttraumatic stress symptoms. The fully adjusted model predicted 31.31% of the variance in symptoms of posttraumatic stress. Racial discrimination (B = 0.01; *p* = .006) and gender discrimination (B = 0.11; *p* = .013) remained significant predictors of higher posttraumatic stress in the adjusted model. Other significant predictors of higher posttraumatic stress included greater social support needs, younger age, having a preexisting mental health diagnosis, having a family member or close friend diagnosed with COVID-19, certain professions, and working more hours during the pandemic.

**Burnout symptoms**. Perceived racial discrimination (B = 0.02; *p* = .001) and gender discrimination (B = 0.50; *p* < .001) predicted burnout symptoms in unadjusted models. The adjusted model explained 29.43% of the variance in burnout symptoms. Gender discrimination, but not racial discrimination, remained a significant predictor of higher burnout symptoms in the adjusted model (B = 0.24; *p* = .001). Other significant predictors of higher burnout symptoms included greater social support needs, younger age, higher household income, certain professions, and working more hours during the pandemic. Compared with White HCWs, Black HCWs had lower symptoms of burnout.

### 3.3. Indirect Effects

**Figure 1:**
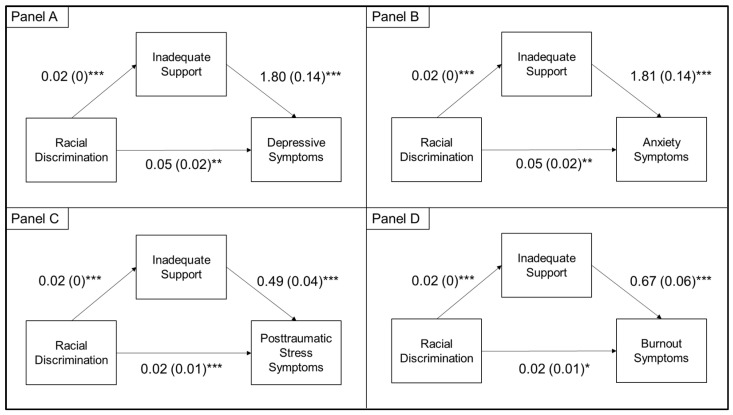
Indirect effects of racial discrimination on symptoms of depression (Panel **A**), anxiety (Panel **B**), posttraumatic stress (Panel **C**), and burnout (Panel **D**) via inadequate social support. The indirect effects analysis controlled for age, gender, race/ethnicity, marital status, profession, pre-pandemic mental health diagnosis, income, family/friend being diagnosed with COVID-19, childcare needs, housing change, frontline status, change in roles, change in work hours, and gender discrimination. * *p* < .05 and ≥ .01; ** *p* < .01 and ≥ .001; *** *p* < .001.

**Figure 2:**
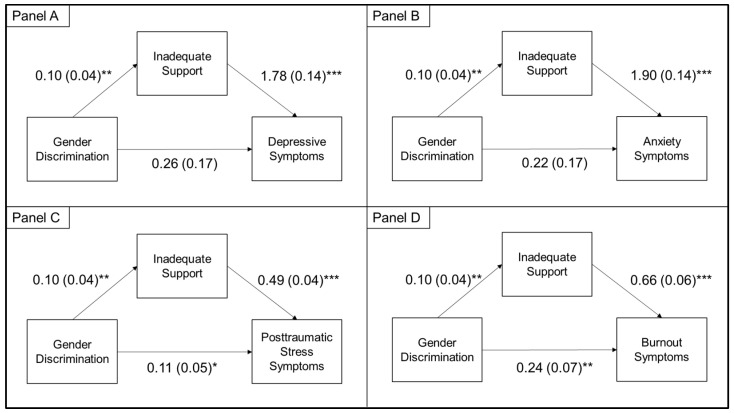
Indirect effects of gender discrimination on symptoms of depression (Panel **A**), anxiety (Panel **B**), posttraumatic stress (Panel **C**), and burnout (Panel **D**) via inadequate social support. The indirect effects analysis controlled for age, gender, race/ethnicity, marital status, profession, pre-pandemic mental health diagnosis, income, family/friend being diagnosed with COVID-19, childcare needs, housing change, frontline status, change in roles, change in work hours, and racial discrimination. * *p* < .05 and ≥ .01; ** *p* < .01 and ≥ .001; *** *p* < .001.

The indirect effects of racial discrimination (Figure 1) and gender discrimination (Figure 2) on mental health outcomes through inadequate social support were statistically significant for all mental health outcomes. In fully adjusted models, the indirect effects of racial discrimination on mental health outcomes through inadequate social support was estimated at 0.04 (95%CI: 0.02, 0.06) for depressive symptoms, 0.04 (95%CI: 0.02, 0.06) for anxiety symptoms, 0.01 (95%CI: 0.01, 0.02) for posttraumatic stress symptoms, and 0.01 (0.01, 0.02) for burnout symptoms. The indirect effects of gender discrimination on mental health outcomes through inadequate social support was estimated at 0.18 (95%CI: 0.04, 0.34) for depressive symptoms, 0.20 (95%CI: 0.05, 0.35) for anxiety symptoms, 0.05 (95%CI: 0.01, 0.09) for posttraumatic stress symptoms, and 0.07 (95%CI: 0.02, 0.12) for burnout symptoms.

## 4. Discussion

In our survey study including 997 HCWs, racial discrimination predicted symptoms of depression, anxiety, and posttraumatic stress after adjusting for gender discrimination, pandemic-related stressors, and demographic characteristics. Further, gender discrimination predicted symptoms of burnout and posttraumatic stress after adjusting for racial discrimination, pandemic-related stressors, and demographic characteristics. Black HCWs had the highest reports of racial discrimination among all racial/ethnic groups. Our findings underscore the substantial adverse effects of discrimination on HCW wellbeing, even during times of significant competing stressors. As pandemic-related stressors begin to wane in the advent of COVID-19 recovery, hospitals should continue to support HCW wellbeing by developing and bolstering diversity and inclusion initiatives, as well as recruiting and supporting the retention and professional development of HCWs of color and female HCWs.

Few studies have assessed discrimination as a risk factor for adverse mental health outcomes among HCWs, and most focus on physicians and physicians-in-training. Studies have identified that racial and gender discrimination were associated with increased risk of burnout among physicians [33] and depression among medical students [34]. Discrimination was also associated with anxiety and depression in a study including nurses in London [35]. The negative effects of discrimination on mental health may be more pronounced for female HCWs of color; for example, one study found that female HCWs of color who reported both racial and gender discrimination were more likely to experience burnout compared with male HCWs of color [7]. However, our study did not find a significant interaction effect of race, gender, and discrimination on mental health outcomes, perhaps due to the relatively small proportions of HCWs of color in our sample. Nonetheless, our study adds to these findings by emphasizing the ongoing and persistent negative effects of racial and gender discrimination on HCW wellbeing even during times of other competing stressors, i.e., social and work stressors associated with the COVID-19 pandemic.

Although Black HCWs reported heightened racial discrimination compared with all other racial/ethnic groups in our study, they had less severe depression, anxiety, and burnout symptoms compared with White HCWs in models that adjusted for discrimination and other covariates. As discrimination was a significant predictor of these outcomes, this finding seemingly contradicts the logic that heightened experiences of racial discrimination among Black HCWs would translate to heightened adverse mental health outcomes. Other studies have also identified that Black HCWs had lower or similar prevalence of adverse mental health outcomes compared with White HCWs [2,36,37,38], a phenomenon consistent with what has been termed the Black-White mental health paradox [39]. One potential explanation could be that stigma prevented Black HCWs from reporting the severity of mental health symptoms [40]. There might be other mechanisms of resiliency among Black HCWs that can explain this association, such as positive coping strategies [41]. For example, a recent national survey study on stress during the COVID-19 pandemic identified that, compared with White HCWs, Black and Latinx HCWs experienced less severe burnout and heightened meaning and purpose, suggesting that positive coping could have protected against symptoms of burnout, including those that were heightened by their experiences of racial discrimination [38]. Furthermore, John Henryism, a high-energy coping style to manage psychosocial stressors such as discrimination, could explain this finding. John Henryism has been associated with lower depressive symptoms at the expense of physical health among Black people [42,43,44]. However, another study found that John Henryism did not modify the relationship between discrimination and depression among Black people [45]. Additional studies are warranted to further explore factors that could buffer the impact of racial discrimination on adverse mental health outcomes among minority HCWs, such as by further evaluating meaning-making and John Henryism as moderators.

We also identified that discrimination had indirect effects on mental health outcomes through inadequate social support. Our findings support the social causation model, which theorizes that experiencing discrimination damages social connections, leading to heightened psychological distress [9,10]. Other studies have also identified that race-based and mental illness-based discrimination had indirect effects on mental health outcomes through social support [10,46,47]. Although no studies to our knowledge have tested this model among HCWs, qualitative studies have identified that minority and female HCWs face discrimination in terms of social exclusion and isolation [30,48] that could prevent them from accessing the mental health benefits of social support. For example, in a qualitative study, Black physicians reported being assumed to be housekeeping and maintenance staff by their colleagues due to racial discrimination, which subsequently made them feel “invisible” and broke social ties with colleagues within the hospital [29]. Given the cross-sectional nature of our survey, longitudinal studies are warranted to further elucidate this potential pathway and others.

Our findings have implications for HCW wellbeing programs, including those that were implemented throughout the COVID-19 pandemic. These initiatives should integrate anti-racism and anti-sexism education and facilitate social support and belonging for minority and female HCWs. Furthermore, hiring and recruiting more racially/ethnically diverse HCWs could improve social support among minority HCWs and mitigate adverse mental health outcomes.

This study has some notable limitations. Although our sample had similar demographic characteristics of HCWs in the US in terms of race/ethnicity and gender [49,50], our convenience sampling approach limits the generalizability of the prevalence of mental health outcomes among all HCWs in the US. Our use of brief, self-report inventories was a further limitation, especially as two of the mental health measures had relatively low internal consistency (i.e., the PC-PTSD and two-item Maslach Burnout Inventory). Further, we excluded four HCWs who identified as non-binary or transgender due to insufficient statistical power to identify differences by gender identity; future studies should purposively recruit gender minority HCWs to understand their experiences of discrimination. Our analysis on the indirect effects of discrimination on mental health outcomes via social support assumes that low social support precedes adverse mental health outcomes (i.e., social causation); however, psychological distress might lead to decreases in perceived social support through social selection, possibly heightening perceptions of discrimination as well [51]. The cross-sectional nature of our survey limited our ability to test whether social causation, social selection, or both were at play; thus, our analyses of indirect effects should be reproduced in longitudinal studies that are better positioned to assess causality.

## 5. Conclusions

We identified that racial and gender discrimination predicted psychological distress symptoms above and beyond individual- and pandemic-related risk factors. We also identified that discrimination might increase the risk for adverse mental health outcomes through unmet social support needs. Future studies are warranted that explore racial and gender discrimination among HCWs and strategies to mitigate adverse mental health effects.

## Figures and Tables

**Table 1:** Participant characteristics.

Characteristics	*n* (%) or *Mean* (*SD*)
Demographic characteristics	
Age	38.22 (11.77)
Pre-pandemic mental health diagnosis	269 (26.98%)
Gender	
Male	285 (28.59%)
Female	712 (71.41%)
Race/ethnicity	
White	688 (69.01%)
Asian	148 (14.84%)
Black	86 (8.63%)
Latinx	73 (7.32%)
Other *	21 (2.11%)
Marital status	
Married	552 (55.37%)
Single	392 (39.32%)
Divorced/widowed	53 (5.32%)
Household income	
<$10,000	61 (6.12%)
$10,000 to $24,999	21 (2.11%)
$25,000 to $49,999	62 (6.22%)
$50,000 to $74,999	173 (17.35%)
$75,000 to $99,999	114 (11.43%)
$100,000 to $149,999	137 (13.74%)
$150,000 to $199,999	110 (11.03%)
$200,000 to 299,999	130 (13.04%)
>$300,000	189 (18.96%)
Profession	
Physician	318 (31.90%)
Trainee	280 (28.08%)
Nurse	125 (12.54%)
Health technician	76 (7.62%)
Physician, nursing, medical assistant	47 (4.71%)
Other clinical	86 (8.63%)
Other non-clinical	65 (6.52%)
**Pandemic-related social factors**	
Family/friend contracted COVID-19	580 (58.17%)
Any housing change	94 (9.43%)
Childcare needs among those with a child (*n* = 258)	
Does not need more childcare support	75 (29.07%)
Needs a little more childcare support	90 (34.88%)
Needs a lot more childcare support	93 (36.05%)
Support needs	
None	296 (29.69%)
A little	262 (26.28%)
Some	284 (28.49%)
A lot	155 (15.55%)
**Pandemic-related work factors**	
Frontline status	
None	330 (33.10%)
Indirect	123 (12.34%)
Direct	544 (54.56%)
Change in hours	
Same	636 (63.79%)
Less	82 (8.22%)
More	279 (27.98%)
Roles changed	452 (45.34%)
**Racial Discrimination**	
Overall	23.65 (8.25)
Race/ethnicity	
White	21.21 (4.77) _b_
Asian	27.26 (9.91) _a,b_
Black	33.96 (12.79) _a_
Latinx	27.20 (10.18) _a,b_
Other *	26.52 (10.96)
**Gender Discrimination**	
Overall	1.65 (0.92)
Gender	
Female	1.85 (0.03) _c_
Male	1.16 (0.05)

Abbreviations: SD, standard deviation. * Other race/ethnicity includes American Indian/Alaska Native, Native Hawaiian/Pacific Islander, and unspecified. For pairwise comparisons with Bonferroni-corrected *p*-values < .05, _a_ represents significantly higher than White participants, _b_ represents significantly lower than Black participants, and _c_ represents significantly higher than men.

**Table 2:** Unadjusted linear regression models for discrimination and mental health.

Characteristics	Depressive Symptoms	Anxiety Symptoms	Posttraumatic Stress Symptoms	Burnout Symptoms
	*B* (SE)	*B* (SE)	*B* (SE)	*B* (SE)
**Discrimination**				
Racial/ethnic discrimination	0.07 (0.02) ***	0.07 (0.02) ***	0.03 (0.01) ***	0.02 (0.01) **
Gender discrimination	0.91 (0.16) ***	0.90 (0.16) ***	0.31 (0.04) ***	0.50 (0.07) ***
**Demographic characteristics**				
Age	−0.01 (0.01)	−0.04 (0.01) **	−0.02 (0.01) ***	−0.02 (0.01) ***
Gender				
Male (Reference)	1.00	1.00	1.00	1.00
Female	1.55 (0.33) ***	1.79 (0.33) ***	0.45 (0.09) ***	0.62 (0.14) ***
Pre−pandemic mental health diagnosis	2.84 (0.33) ***	2.65 (0.33) ***	0.53 (0.09) ***	0.59 (0.14) ***
Race/ethnicity				
White (Reference)	1.00	1.00	1.00	1.00
Asian	−0.46 (0.43)	−0.61 (0.43)	−0.10 (0.12)	−0.15 (0.18)
Black	−0.85 (0.54)	−0.88 (0.55)	0.04 (0.15)	−0.58 (0.23) *
Latinx	0.70 (0.58)	0.62 (0.59)	0.23 (0.16)	0.29 (0.29)
Other	−1.25 (1.05)	−0.74 (1.06)	−0.41 (0.29)	−0.58 (0.44)
Marital status				
Married (Reference)	1.00	1.00	1.00	1.00
Single	1.09 (0.31) **	1.02 (0.32) **	0.32 (0.09) ***	0.32 (0.13) *
Divorced/widowed	1.89 (0.68) **	0.16 (0.69)	0.24 (0.19)	−0.17 (0.29)
Household income	−0.17 (0.06) **	−0.11 (0.06)	−0.04 (0.02) *	0.01 (0.03)
Profession				
Physician (Reference)	1.00	1.00	1.00	1.00
Trainee	0.45 (0.39)	0.21 (0.39)	0.13 (0.11)	0.20 (0.16)
Nurse	1.76 (0.50) ***	2.31 (0.50) ***	0.66 (0.14) ***	0.73 (0.21) **
Health technician	1.67 (0.60) **	2.06 (0.60) **	0.33 (0.17) *	0.23 (0.25)
Physician, nursing, medical assistant	2.17 (0.74) **	2.67 (0.74) ***	0.65 (0.20) **	0.69 (0.31) *
Other clinical	1.03 (0.57)	0.43 (0.57)	0.19 (0.16)	−0.39 (0.24)
Other non-clinical	1.69 (0.64) **	2.08 (0.64) **	0.60 (0.18) **	0.16 (0.27)
**Pandemic-related social factors**				
Family/friend contracted COVID-19	0.86 (0.31) **	1.19 (0.31) ***	0.41 (0.08) ***	0.18 (0.13)
Any housing change	2.02 (0.51) ***	1.38 (0.52) **	0.53 (0.14) ***	0.59 (0.22) **
Childcare needs	0.51 (0.24) *	0.84 (0.24) **	0.18 (0.07) **	0.37 (0.10) ***
Support needs	2.06 (0.21) ***	2.21 (0.13) ***	0.61 (0.04)	0.86 (0.05) ***
**Pandemic-related work factors**				
Frontline status				
None (Reference)	1.00	1.00	1.00	1.00
Indirect	0.16 (0.50)	−0.09 (0.51)	0.15 (0.14)	0.26 (0.21)
Direct	0.13 (0.33)	0.16 (0.34)	0.23 (0.09) *	0.64 (0.14) ***
Change in hours				
Same (Reference)	1.00	1.00	1.00	1.00
Less	0.13 (0.55)	−0.43 (0.56)	−0.09 (0.15)	−0.20 (0.23)
More	1.45 (0.34) ***	1.07 (0.34) **	0.47 (0.47) ***	1.15 (0.14) ***
Roles changed	0.91 (0.30) **	0.81 (0.30) **	0.41 (0.08) ***	0.57 (0.13) ***

Abbreviations: B, unstandardized beta coefficient; SE, standard error. Other race/ethnicity includes American Indian/Alaska Native, Native Hawaiian/Pacific Islander, and unspecified. * *p* < .05 and ≥ .01; ** *p* < .01 and ≥ .001; *** *p* < .001.

**Table 3:** Adjusted linear regression models for discrimination and mental health.

Characteristics	Depressive Symptoms	Anxiety Symptoms	Posttraumatic Stress Symptoms	Burnout Symptoms
	*B* (SE)	*B* (SE)	*B* (SE)	*B* (SE)
**Discrimination**				
Racial/ethnic discrimination	0.04 (0.02) *	0.05 (0.02) **	0.01 (0.01) **	0.01 (0.01)
Gender discrimination	0.25 (0.17)	0.20 (0.17)	0.11 (0.05) *	0.24 (0.07) **
**Demographic characteristics**				
Age	0.03 (0.02) *	−0.02 (0.02)	−0.01 (0) **	−0.01 (0.01) *
Gender				
Male (Reference)	1.00	1.00	1.00	1.00
Female	−0.02 (0.34)	0.03 (0.33)	−0.07 (0.09)	0.04 (0.14)
Pre-pandemic mental health diagnosis	2.01 (0.31) ***	1.69 (0.30) ***	0.23 (0.08) **	0.21 (0.13)
Race/ethnicity				
White (Reference)	1.00	1.00	1.00	1.00
Asian	−0.15 (0.41)	−0.35 (0.40)	−0.11 (0.11)	−0.28 (0.17)
Black	−1.52 (0.53) **	−1.42 (0.53) **	−0.13 (0.15)	−0.62 (0.22) **
Latinx	0.07 (0.52)	0.03 (0.51)	0.04 (0.14)	0.12 (0.22)
Other	−1.45 (0.91)	−0.55 (0.90)	−0.40 (0.25)	−0.55 (0.38)
Marital status				
Married (Reference)	1.00	1.00	1.00	1.00
Single	0.67 (0.36)	0.66 (0.36)	0.12 (0.10)	0.22 (0.15)
Divorced/widowed	1.11 (0.62)	−0.14 (0.61)	0.18 (0.17)	0.03 (0.26)
Household income	0.01 (0.09)	0.16 (0.09)	0.03 (0.02)	0.13 (0.04) **
Profession				
Physician (Reference)	1.00	1.00	1.00	1.00
Trainee	−0.04 (0.47)	−0.48 (0.47)	−0.09 (0.13)	0.39 (0.25)
Nurse	0.77 (0.49)	1.42 (0.48) *	0.47 (0.13) ***	0.70 (0.20) **
Health technician	1.12 (0.59)	1.77 (0.58) **	0.29 (0.16)	0.47 (0.25)
Physician, nursing, medical assistant	0.89 (0.70)	1.49 (0.69) *	0.40 (0.19) *	0.77 (0.29) *
Other clinical	0.70 (0.58)	0.20 (0.58)	0.21 (0.16)	0.05 (0.24)
Other non-clinical	0.83 (0.63)	1.33 (0.62) *	0.43 (0.17) *	0.33 (0.26)
**Pandemic-related social factors**				
Family/friend contracted COVID-19	0.36 (0.27)	0.65 (0.27) *	0.23 (0.07) **	0.01 (0.11)
Any housing change	0.60 (0.47)	−0.11 (0.46)	0.02 (0.13)	−0.04 (0.20)
Childcare needs	0.05 (0.23)	0.22 (0.23)	0.01 (0.06)	0.07 (0.10)
Support needs	1.77 (0.14) ***	1.89 (0.14) ***	0.49 (0.04) ***	0.65 (0.06) ***
**Pandemic-related work factors**				
Frontline status				
None (Reference)	1.00	1.00	1.00	1.00
Indirect	−0.09 (0.44)	−0.31 (0.44)	0.08 (0.12)	0.02 (0.19)
Direct	−0.20 (0.33)	−0.43 (0.32)	0.07 (0.09)	0.18 (0.14)
Change in hours				
Same (Reference)	1.00	1.00	1.00	1.00
Less	0.18 (0.51)	−0.26 (0.50)	−0.11 (0.14)	−0.26 (0.21)
More	0.70 (0.32) *	0.32 (0.32)	0.23 (0.09) **	0.75 (0.14) ***
Roles changed	−0.24 (0.29)	−0.21 (0.29)	0.11 (0.08)	0.15 (0.12)

Abbreviations: B, unstandardized beta coefficient; SE, standard error. Other race/ethnicity includes American Indian/Alaska Native, Native Hawaiian/Pacific Islander, and unspecified. * *p* < .05 and ≥ .01; ** *p* < .01 and ≥ .001; *** *p* < .001.

## Data Availability

The data presented in this study are available on request from the corresponding author.
